# The Physical Clogging of the Landfill Leachate Collection System in China: Based on Filtration Test and Numerical Modelling

**DOI:** 10.3390/ijerph15020318

**Published:** 2018-02-12

**Authors:** Yili Liu, Weixin Sun, Bing Du, Jianguo Liu

**Affiliations:** Key Laboratory for Solid Waste Management and Environment Safety, Ministry of Education of China, School of Environment, Tsinghua University, Beijing 100084, China; ylliu@foxmail.com (Y.L.); nzfinal2008@sina.com (W.S.); w.dubing@gmail.com (B.D.)

**Keywords:** landfill leachate, leachate collection system (LCS), clogging, particulate matter, numerical simulation

## Abstract

Clogging of the leachate collection system (LCS) has been a common operation problem in municipal solid waste (MSW) landfills in China, which can result in high water levels that threaten the safety of landfill operations. To determine the cause of failure in an LCS, raw leachate from a municipal solid waste transfer station was collected and the high content of particulate matter was characterized. Based on the parameters obtained in a filtration test, a numerical simulation was performed to estimate the influence of particle deposition on drainage system clogging. The results showed that LCSs were confronted with the risk of clogging due to the deposition of particulate matter resulting from the higher concentration of total suspended solids (TSS level > 2200 mg L^−1^) and larger particle size (>30% TSS particles > 15 μm) in the leachate. On one hand, the non-woven geotextile, as the upper layer of the LCS, retained most particulate matter of large diameters, reducing its hydraulic conductivity to approximately 10^−8^ to 10^−9^ m s^−1^ after 1–2 years of operation and perching significant leachate above it (0.6–0.7 m). On the other hand, the geotextile prevented the gravel layer from physically clogging and minimized the leachate head above the bottom liner. Therefore, the role of geotextile should be balanced to optimize the LCS in MSW landfills in China.

## 1. Introduction

In 2016, more than 203 million tons of municipal solid waste (MSW) were generated and 60.3% of it was disposed of by landfilling in urban areas of China [1]. Different from the “dry tomb” landfills in the developed countries, a great quantity of water accumulates in the MSW landfills in China and is difficult to be drained. Leachate head is quite high, ranging from several to dozens of meters in MSW landfills in China [2]. As a result, leachate saturates the void in the waste and reduces the shear strength of the landfill body, which is considered to be the main factor causing the failure of landfill slope [3]. Meanwhile, the leachate can block landfill gas (LFG) collection channels and reduce the efficiency of collection systems, which would promote the fugitive emissions of LFG in return [4,5]. Moreover, high leachate head raises the potential for leachate leakage and poses a high risk of groundwater contamination [6]. Therefore, “water management” is an important issue in the MSW landfills in China.

Until now, the structure of leachate collection systems (LCSs) has been improving over several generations. Current systems involve continuous spreading of the drainage layer at the bottom of the landfill site and isolating drainage gravel from waste using geotextile. In addition, drains are arranged at intervals in such systems [7]. However, the newly specified LCS does not entirely solve the problem of high leachate levels in China, because LCS clogging results in a reduction in leachate drainage. For example, the field hydraulic conductivity of the LCS was tested as low as 10^−8^ m s^−1^ at the Laohukeng sanitary landfill in Shenzhen, China [8].

Biofilm growth, mineral precipitation, and suspended particulate matter deposition are the main mechanisms of LCS clogging [9]. In a batch synthetic and real leachate irrigating experiment, Fleming and Rowe determined that CO_3_^2−^ from the microbial degradation of volatile fatty acids in leachate binds to Ca^2+^ in leachate to form calcium carbonate as the primary driver of LCS clogging [10]. Meanwhile, by column experiment, Rowe et al. pointed out that the column irrigated with real landfill leachate reduced the porosity of the drainage layer by 24% more than that with the synthetic leachate without suspended particulate matter) [11]. This result indicated the high contribution of particulate matter to LCS clogging.

By systematic experiments and numerical simulation, Rowe et al. calculated the leachate mound depth in the LCS and predicted its service life. The results suggested that the LCS could be effective for decades in most situations [12]. Nevertheless, our field investigations found that the LCS in MSW landfills in China nearly failed within several months due to fast clogging, which was most likely a result of particulate matter deposition.

Compared with developed countries, the higher proportion of food waste in China results in the generation of more leachate per ton of waste [13,14] and leads to a higher concentration of organic suspended particulate matter [15], which further favors physical clogging of LCSs in the landfills in China. However, few studies have been done on the influence of particulate matter deposition on LCS clogging.

Moreover, previous studies always used the leachate samples collected from the leachate equalization basin in the landfills [16,17,18]. Herein, the collected leachate had been filtered and a large amount of particulate matter had already been retained by the LCS. Also, the characteristics of the particulate matter changed a lot between the “filtered” and the raw leachate. As a result, these experiments underestimated the contribution of particulate matter to LCS clogging.

In this study, raw leachate from an MSW transfer station was analyzed to characterize the particulate matter, and a filtration test was conducted to evaluate the retention of particulate matter by geotextile, then a mathematical model was established and solved numerically to simulate the physical clogging development in LCSs and leachate accumulation in MSW landfills in China.

## 2. Methods

### 2.1. Filtration Test

The raw leachate was collected from the leachate storage bunker in the Xiaowuji MSW transfer station in Beijing. Without filtration by the LCS, similar to that collected in MSW landfills, the leachate kept the original particulate matter.

During the filtration test, the raw leachate was slowly irrigated through the 2-mm-thick (200 g m^−2^) geotextile, and the negative pressure suction was applied when the hydraulic conductivity was lower than 10^−6^ m s^−1^. When testing the hydraulic conductivity, two methods of falling head and constant head were duly adopted according to the filtration velocity [19]. The properties of particulate matter in the filtered leachate such as TSS (total suspended solids), VSS (volatile suspended solids) and FSS (fixed/inorganic suspended solids) were tested using a gravimetric measurement of the residue retained on a 0.45 μm glass fiber filter dried at 105 and 550 °C, respectively [20]. Meanwhile, a laser particulate matter size analyzer (LS13320, Beckman, Redlands, CA, USA) was used to measure the diameter distribution functions.

By inverting using the Kozeny–Carman equation (in Section 3.1), the equivalent porosity of the geotextile could be estimated at a certain hydraulic conductivity. Then, combining the filtration test results together, the change in retention ratio (*η_f_*) for each particle size in different geotextile porosities could be calculated.

### 2.2. Numerical Simulation

#### 2.2.1. Physical Model

**Figure 1:**
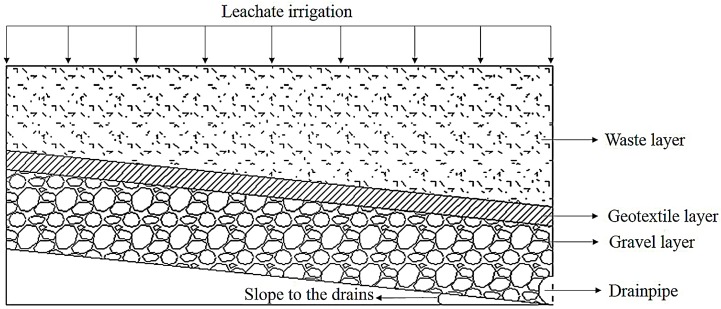
Cross-sectional diagram of the leachate collection system.

When leachate filters through the drainage layer, particulate matter is retained and fills pores. This results in a drastic decrease in hydraulic conductivity and accumulation of leachate in the modeling zone. Figure 1 shows a cross-section of an LCS. Leachate uniformly filters through the waste layer. In this study, based on the average investigation result of serval typical landfills located in different parts of China, the flow-in flux was set to 1 m^3^ m^−2^ year^−1^. Then, leachate filters through the LCS, which consists of a geotextile layer, gravel layer, and drainpipe. Although the porosity of the waste layer decreases with MSW degradation, it was set to 0.375 for simplification [21]. According to the *Technical Code for Sanitary Landfill Site of Municipal Solid Waste* (GB 50869-2013), the thickness of the non-woven geotextile layer was set to 2 mm (200 g m^−2^) with a porosity of 0.9 and initial hydraulic conductivity of 10^−3^ m s^−1^. The thickness of the gravel layer was 300 mm, and the gravel diameter was 20 mm with a porosity of 0.36 and initial hydraulic conductivity of 0.037 m s^−1^. According to the specification, the slope to the drains was set to 2% and the drainage length was 25 m, as proposed by Wan [22]. The drainpipe could be regarded as an open boundary with a diameter of 100 mm. The other boundaries were considered as free flux boundaries.

#### 2.2.2. Mathematical Model

The mathematical relationships among the parameters were built according to the physical model and the governing equations were listed as follows.

##### Water Movement

Drainage layer clogging will elevate the free leachate head progressively. To avoid boundary changes caused by fluctuation in the water surface, a saturated–unsaturated transient governing equation was applied as the basic equation to simulate the flow of leachate [23]. Pressure head, *h*, was considered as the dependent variable in the Equation (1).

(1) $$C\left(\theta \right)\frac{\partial h}{\partial t}=\frac{\partial }{\partial x}\left(K_{XX}\left(h\right)\frac{\partial h}{\partial x}\right)+\frac{\partial }{\partial z}\left(K_{zz}\left(h\right)\frac{\partial h}{\partial z}\right)+\frac{\partial \left(K_{zz}\left(h\right)\right)}{\partial z}+W$$

Here, *h* is the pressure head (m), *θ* is the pore water content (dimensionless) where 0 < *θ* < *n*, *n* is the porosity (dimensionless), *K*(*h*) is the non-saturated hydraulic conductivity tensor (m s^−1^), *W* is the term of source and sink (s^−1^) and *C*(*θ*) is the water capacity (m^−1^). In addition, *t* is the time and *x*, *z* represent the horizontal and vertical direction, respectively. The relationship between the hydraulic conductivity and saturation could be calculated using the empirical Formulas (2)–(4) of van Genuchten [24,25]:

(2) $$\theta =\theta_{r}+S_{e}\times \left(\theta_{S}-\theta_{r}\right)$$

(3) $$S_{e}=\frac{1}{{\left(1+{\left|A\times H_{p}\right|}^{N}\right)}^{M}}$$

(4) $$K_{\left(h\right)}=K_{s}\times {S_{e}}^{\frac{1}{2}}\times {\left[1-{\left(1-{S_{e}}^{\frac{1}{M}}\right)}^{M}\right]}^{2}$$

Here, *θ_r_* and *θ_s_* are the residual and saturated volumetric water contents (dimensionless), *S_e_* is the effective saturation (dimensionless), constants *A*, *N*, and *M* are specified to a particular medium type, *H_p_* is the posed in terms of pressure head (m) and *K_s_* is the saturated hydraulic conductivity of the porous medium (m s^−1^). In addition, if *H_p_* ≥ 0, then *S_e_* = 1.

##### Particulate Matter Motion

Equation (5) describes the movements of particulate matter with water motion under saturated–unsaturated conditions.

(5) $$\frac{\partial \theta C}{\partial t}=\frac{\partial }{\partial x}\left(\theta D_{xx}\frac{\partial C}{\partial x}+\theta D_{xz}\frac{\partial C}{\partial z}\right)+\frac{\partial }{\partial z}\left(\theta D_{zx}\frac{\partial C}{\partial x}+\theta D_{zz}\frac{\partial C}{\partial z}\right)-\frac{\partial \theta u_{x}C}{\partial x}-\frac{\partial \theta u_{z}C}{\partial z}+I$$

Here, *C* is the particulate matter concentration in the liquid phase (kg m^−3^), *D_xx_*, *D_xz_*, *D_zx_*, and *D_zz_* correspond to the coordinate components of the hydrodynamic dispersion coefficient tensor *D* (m^2^ s^−1^), and *I* is the amount of particulate matter that decreased as a result of clogging and filtration of the drainage medium (kg m^−3^ s^−1^).

In the LCS, the particulate matter concentration decreased because of the deposition of suspended particulate matter in the upper layer. Per unit time, the amount of adsorptive particulate matter in liquid was proportional to the concentration of suspended particulate matter and fluid velocity. Therefore, the filtration coefficient was defined as *λ* (m^−1^) [26,27] and resulted in the Equation (6):

(6) $$\frac{\partial C}{\partial t}=\theta \lambda \nu C$$

Here, *v* is the pore velocity in the porous medium (m s^−1^).

Based on the mass balance, the quality increase of sedimentary particulate matter concentration *C_s_* (kg m^−3^) equaled to the total mass loss in the liquid phase within each finite element, then:

(7) $$\frac{\partial C_{s}}{\partial t}=-\theta \frac{\partial C}{\partial t}$$

##### Particulate Matter Filtration Coefficient

The adsorption coefficient was defined as *λ_f_*, (m^−1^) in Equation (8) when particulate matter is irrigated through geotextile [28]:

(8) $$\lambda_{f}=\frac{-ln\left(1-\eta_{f}\right)}{a}$$

Here, *η_f_* is the retention ratio of the particulate matter in the geotextile layer (dimensionless, 0 < *η_f_* < 1), and *a* is the thickness of the geotextile (m).

There is currently inadequate research on *η_f_*. In this study, the retention ratio of geotextile with different porosities for particulate matter of different sizes was confirmed in the filtration test (Section 2.1 and Section 3.1).

When particulate matter filtered through the gravel drainage layer, the filtration coefficient was set to *λ_g_* (m^−1^) (Equation (9)) [9]:

(9) $$\lambda_{g}=\frac{3\left(1-n\right)}{2d_{g}}\eta_{g}$$

Here, *η_g_* is the retention ratio of gravel for particulate matter (dimensionless, 0 < *η_g_*< 1), and *d_g_* is the gravel diameter (m).

Interception, diffusion by Brownian movement, and gravitational sedimentation influenced particulate matter deposition in the gravel layer [29,30], and the retention ratio of the gravel layer could be calculated as Equation (10):

(10) $$\eta_{g}={\left(1-n\right)}^{\frac{2}{3}}\times A_{S}\times N_{Lo}^{\frac{1}{8}}\times N_{R}^{\frac{15}{8}}+3.375\times 10^{-3}\times {\left(1-n\right)}^{\frac{2}{3}}\times A_{S}\times N_{G}^{1.2}\times N_{R}^{-0.4}+4A_{S}^{\frac{1}{3}}\times N_{pe}^{-\frac{2}{3}}$$

Here, *A_s_*, *N_Lo_*, *N_R_*, *N_G_*, and *N_Pe_* are calculated following the R–T method [31], and these parameters represent the flowage, London force, interception, gravitational, and Peclet number, respectively.

##### Coupling Relationship among Parameters

During drainage layer operation, particulate matter deposition led to a reduction in the porosity of porous media. Therefore:

(11) $$n=n_{0}-\frac{C_{s}}{X_{p}\left(1-\varepsilon_{p}\right)}$$

Here, *n*_0_ is the initial porosity, *X_p_* is the real density of particulate matter (kg m^−3^), and *ε_p_* is the inside porosity of packed particulate matter (dimensionless) [32]. By dividing (1 − *ε_p_*), the actual porosity blocked by the particulate matter in the porous material could be assumed. In this study, this value (*ε_p_*) was 0.85, which was confirmed by the measured moisture content of sediment. Previous tests have suggested that the hydraulic conductivity coefficient of non-woven geotextile can be reduced to 10^−10^ m s^−1^ (Section 3.1), while the porosity of the drainage layer affected by clogging could decrease to 0.01–0.05 [20,33]. To avoid non-convergence of the calculations, the minimum hydraulic conductivity was set to 10^−10^ m s^−1^ and the minimum porosity to 0.01.

Assuming all sediment in the pores covers the gravel uniformly, the equivalent diameter of the gravel along with the wrapped particles layer increases as more particulate matter attaches to the porous medium surface. Then, the equivalent diameter could be updated with Equation (12) when calculating the related parameters such as *λ_g_*, *N_R_*, and *N_pe_*:

(12) $$d_{g}=d_{g,0}\times \sqrt{1+\frac{n_{0}-n}{\pi }}$$

The relationship between the saturated hydraulic conductivity of porous media and the porosity of the non-woven geotextile layer could be represented by the Kozeny–Carman Equation (13) [34]:

(13) $$K_{t}=K_{0}\times \frac{n^{3}}{{\left(1-n\right)}^{2}}\times \frac{{\left(1-n_{0}\right)}^{2}}{{n_{0}}^{2}}$$

Here, *K_t_* is the real-time hydraulic conductivity (m s^−1^) and *K*_0_ is the initial hydraulic conductivity of the porous media (m s^−1^).

Meanwhile, in the gravel layer, the specific correlation between the hydraulic conductivity and the porosity was fully studied by Yu and Rowe [35] and it could be described by exponential form (Equation (14)):

(14) $$K_{t}=K_{a}\times e^{K_{b}\times n}$$

Here, *K_a_* = 9.8 × 10^−6^ (m s^−1^) and *K_b_* = 22.9 when *n* > 0.21, and *K_a_* = 2.4 × 10^−8^ (m s^−1^) and *K_b_* = 51.0 when *n* < 0.21 [35]. Although the hydraulic conductivity increases with the enlargement of the gravel size, in this study, the fixed calculation parameters were adopted for simplification.

#### 2.2.3. Solution of the Model

The finite element method was applied to predict the leachate movement in the whole modeling area and the physical clogging inside the LCS. By using the Comsol Multiphysics 5.2a software, the subdivided mesh quantities were set to 10,663, 1900, and 1900, in the waste layer, geotextile layer, and gravel layer, respectively. When calculated, a parallel sparse direct solver (MUMPS) built in the software was applied to perform a fully coupling simulation at each time interval [36]. The time interval was set to 0.001 day initially and the maximum was 1 day.

## 3. Results

### 3.1. Retention Ratio of Geotextiles

The raw leachate samples were tested and the concerned properties are shown in Table 1. 

**Figure 2:**
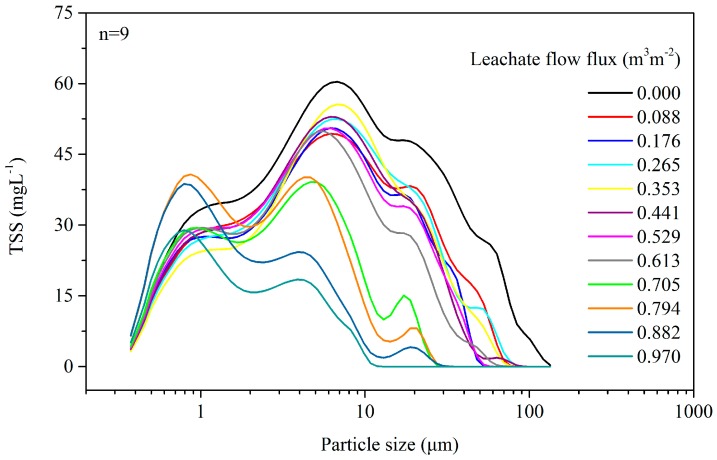
Particulate matter sizes and concentrations in leachate under different filtration fluxes.

**Figure 3:**
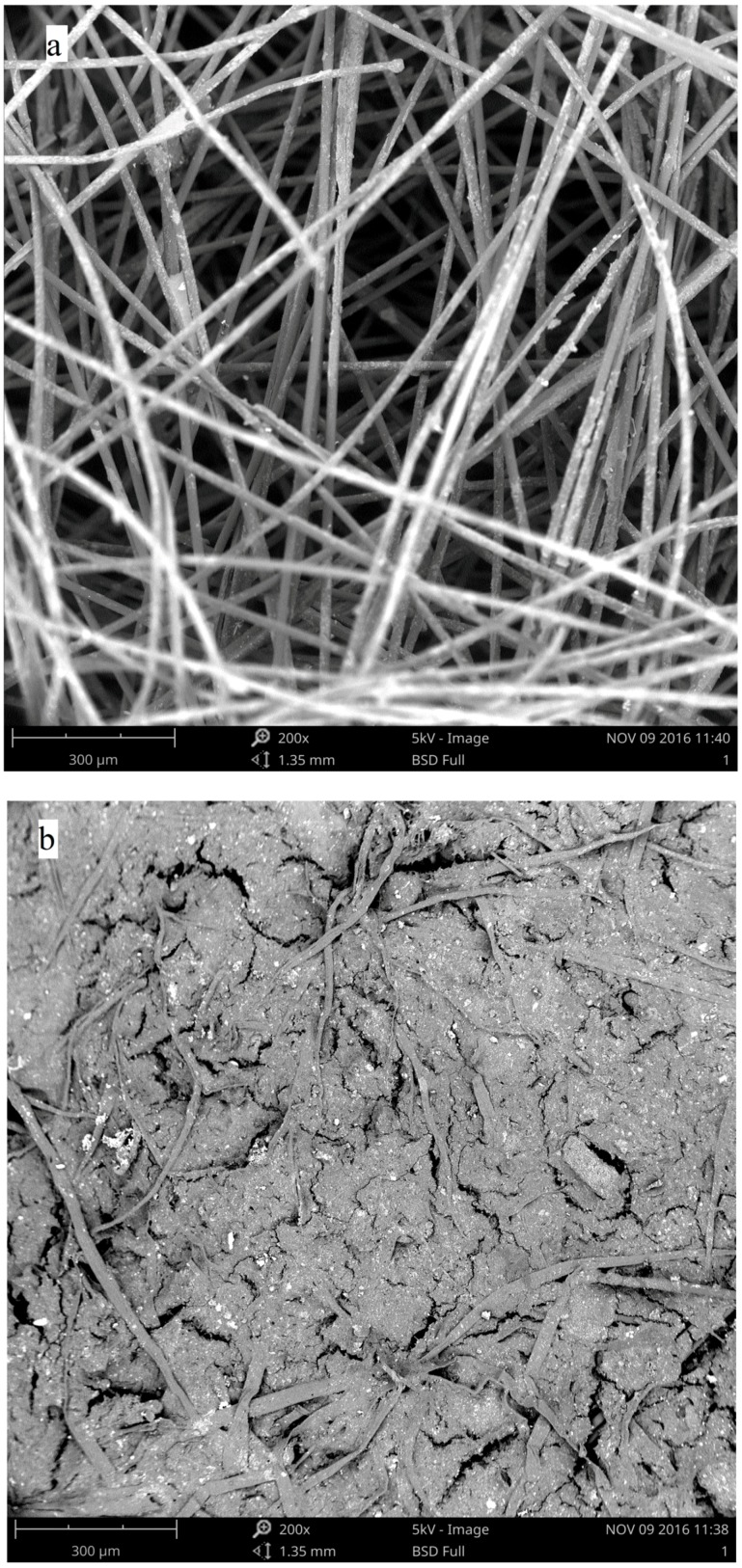
The SEM photos of the geotextile before (**a**) and after (**b**) the filtration test.

Figure 2 presents the size distribution of particulate matter and TSS in raw (flux = 0) and filtrated leachate under different fluxes. In the raw leachate, the TSS concentration was more than 2200 mg L^−1^ and over 30% of it fell in the range of particle size greater than 30 μm. Then, the particle concentration decreased significantly with the higher filtration flux. Meanwhile, the particle size distribution became narrower and the average particle size became smaller. The SEM (scanning electron microscope) photos of the geotextile before and after (flux = 0.97 m^3^ m^−2^) the filtration test show that nearly all the pores were clogged by the particulate matter (Figure 3).

**Figure 4:**
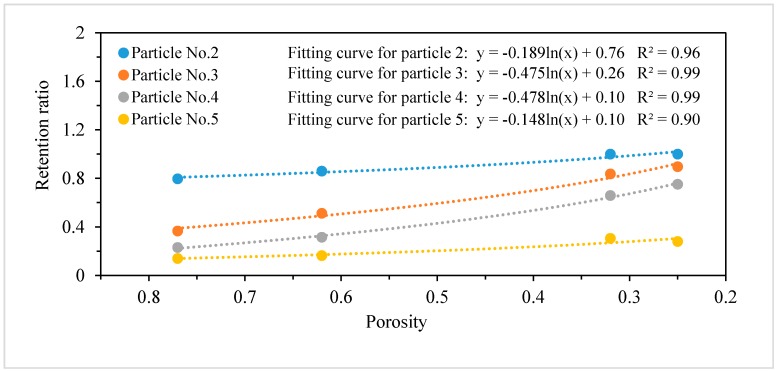
Retention ratio of the geotextile layer for particulate matter of different sizes under different porosities.

By inverting using the Kozeny–Carman Equation (13), the equivalent porosity of the geotextile could be estimated at a certain hydraulic conductivity (Figure S1, the hydraulic conductivity of geotextiles under different filtration fluxes). Then, combining the filtration test results together (Figure 2), the change in retention ratio (*η_f_*) for each particle size in different geotextile porosities could be curve fitted and built into the numerical model (Figure 4). Affected by the limitation of the subsequent finite element calculation, the distribution curve was maximally divided into six segments according to the particulate matter size. Further, six representative sizes of particulate matter were chosen to represent the diameter and concentration of each size group (Table S1).

Moreover, the test results showed that nearly all the particulate matter of the diameter beyond 65 μm (particulate matter No. 1) would be retained by the geotextile at the very beginning (accumulative flux flow < 0.09 m^3^ m^−2^). Therefore, the retention ratio of particulate matter No. 1 was set to 0.999 in the numerical simulation. On the contrary, the retention ratio was relatively lower and abnormal for particulate matter of a diameter of 0.4–1.6 μm (particulate matter No. 6). This phenomenon might be caused by two reasons: first, the pore water velocity accelerated (with the reduction of porosity under the constant flow rate condition) and further lowered the retaining efficiency [37]; second, some particles with large diameters could break apart and lead to an increase in the concentration of this particle size segment. Therefore, to simplify the calculation, the retention ratio for particulate matter No. 6 was set to 0.15.

### 3.2. Clogging Process in the Leachate Drainage Layer

**Figure 5:**
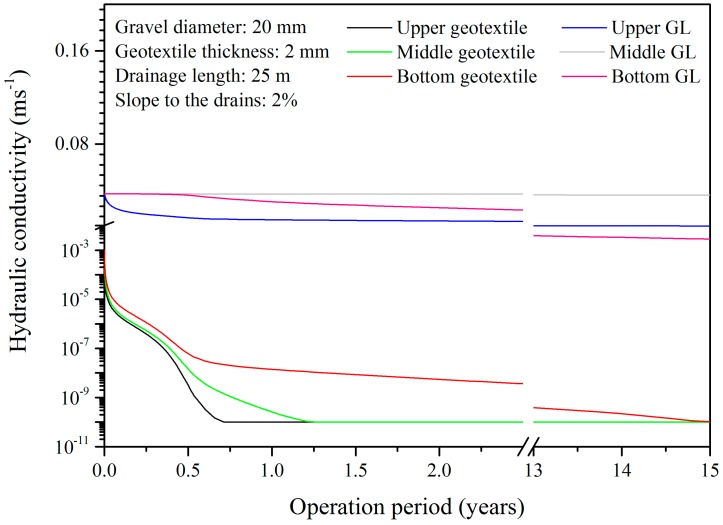
Decrease in hydraulic conductivity in the geotextile and gravel (GL) layers of the leachate collection system (LCS) over time.

The hydraulic conductivity in different positions along the vertical axis of the central drainage system (*x* = 12.5 m) changed over time as shown in Figure 5. The results showed that the surface and middle layers of the geotextile clogged substantially, where the hydraulic conductivities reached the cutoff value (10^−10^ m s^−1^) after 8.5 and 15.1 months of service, respectively. However, the particulate matter had a weaker influence on the lower geotextile level.

Initially, only the large particles could be retained by the clean geotextile. Over time, the hydraulic conductivity and porosity of the upper geotextile declined, which facilitated the retention of the smaller particles and the acceleration of clogging. Under the protection of the surface geotextile layer, the curve of the hydraulic conductivity in the middle and lower geotextile layer showed another tendency for the clogging rate to slow down after half of a year’s operation.

The geotextile performed well in protecting the gravel layer. Over the long operation period (15 years), the hydraulic conductivities of the upper and middle gravel layers decreased by approximately 74.1% and 2.9%, respectively, while the number was about 92.5% for the lower gravel layer. Since the water-holding capacity of the gravel layer was relatively weak, the saturation was highly asymmetric. According to the literatures and the built model in this study, the particulate matter retaining efficiency was positive to the water content in the drainage system [38,39]. Therefore, the saturated bottom region had a higher particulate matter retaining efficiency, which promoted the particulate matter deposition and the clogging development.

### 3.3. Changes in the Leachate Head

Initially, there was no continuous water clogging above the geotextile nor bottom liner, because the drainage system was cleaner, with inflowing leachate infiltrating in a timely manner. As particulate matter deposited gradually within the LCS, the hydraulic conductivity of the geotextile surface decreased, resulting in leachate gradually accumulating in the LCS. The leachate infiltration speed was influenced by the hydraulic pressure gradient and hydraulic conductivity in accordance with Darcy’s law. As the hydraulic conductivity of the LCS gradually decreased, the enhanced leachate depth in the waste layer realized a dynamic balance between the leachate inflow speed and its infiltration capability.

**Figure 6:**
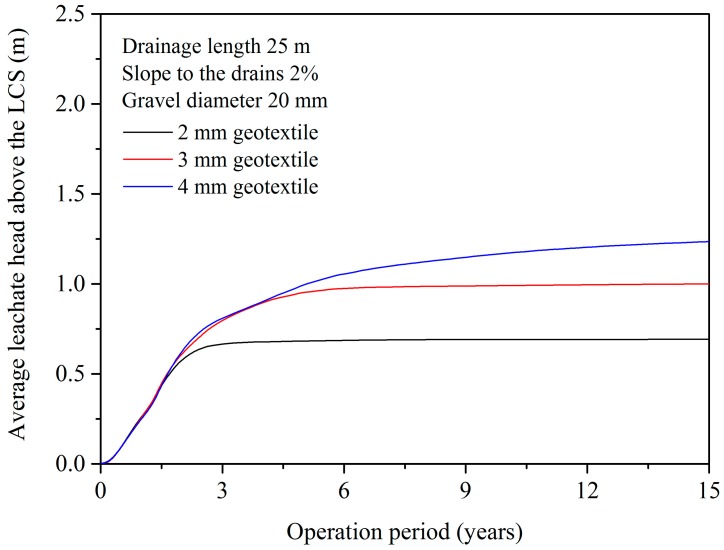
Average leachate head above the LCS.

In the scenario with the 2-mm-thick (200 g m^−2^) geotextile, the inflow speed kept up with the infiltration capacity after approximately 2–3 years, and the upper water level above the geotextile layer leveled off at 0.6–0.7 m (Figure 6). Conversely, thanks to the protection of geotextile, the leachate head on the bottom liner increased slowly and slightly. The leachate mound caused by physical clogging was around 2 cm maximally.

## 4. Discussion

### 4.1. Influence of Leachate Properties

Compared with developed countries, both the high moisture content and large food waste proportion of MSW in China have negative impacts on landfill drainage systems. On the one hand, Yang et al. calculated the leachate output was about 500 kg per ton of disposed waste in China and this was much higher than the 150 kg ton^−1^ estimated by [14,40]. Based on the flux accounting alone, the LCS service life could be shortened to 30%. Moreover, the higher leachate outputs could enhance the saturation of the LCS, which would further assist the clogging [41]. On the other hand, the more food waste generated, the higher the concentration of organic particles with larger particle size in the leachate in China, which could be the leading cause of physical clogging. In Canada, a 6-year mesocosm experiment showed that the hydraulic conductivity reduction of the non-woven geotextile was about 90% (from initial 4.4 × 10^−4^ m s^−1^ to 4.6 × 10^−5^ m s^−1^ in the end) [42]. However, in China, the test and numerical simulation results showed the physical blockage alone could reduce the hydraulic conductivity by 6–7 orders of magnitude to 10^−9^–10^−10^ m s^−1^.

**Figure 7:**
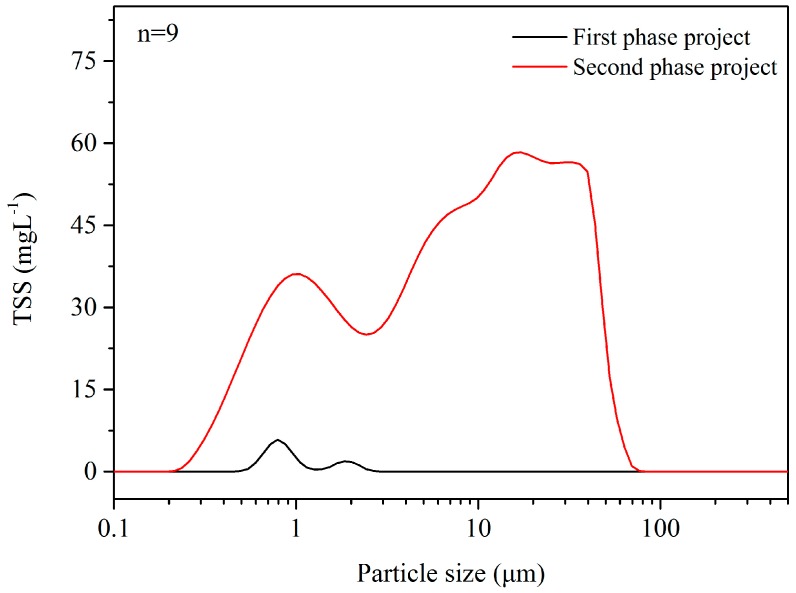
Particle size distribution of leachate from the Wuhan Chen-Jia-Chong landfill.

The analyses in Section 3.2 and Section 3.3 show that particulate matter deposition in drainage systems is a major factor driving the reduction in the drainage capacity of LCSs. This conclusion was confirmed by an investigation of MSW landfill in Wuhan (in the central part of China; the annual precipitation and average temperature were 1260 mm and 16 °C, respectively) carried out by our research group. This MSW landfill had a total storage capacity of 5.3 million cubic meters and disposed of 6000–7000 tons of waste per day. It underwent two phases of operation; the first phase ran for a decade and the height of the landfill body was over 65 m, while the newly built second phase had only been running for a month at the time of the investigation. The LCS for the first phase was an obvious failure and the lateral leakage of leachate appeared in the slopes of the landfill body. Considering the same MSW was disposed of, the particulate matter analysis of the leachate sampled from the different leachate equalization basins of the two phases indicated that most of the generated particulate matter was retained in the first phase (Figure 7). Meanwhile, the comparison of the particle size distribution functions of the leachate from the leachate equalization basin (second phase) and MSW transfer station (Figure 2) suggested that even the new LCS could retain nearly all the particulate matter with a diameter greater than 70 μm.

### 4.2. The Effect of Geotextile

**Figure 8:**
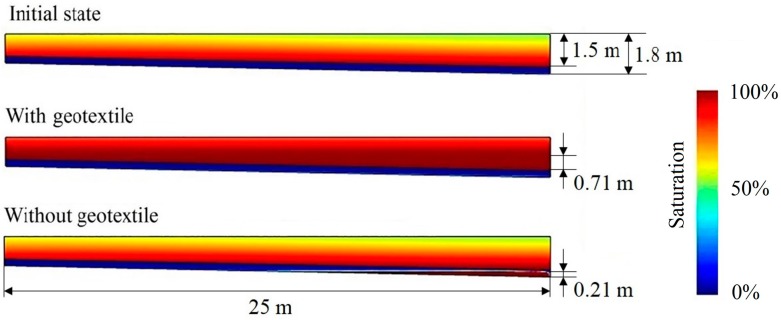
Degree of saturation in the waste layer and LCS (geotextile thickness 2 mm, drainage length 25 m, gravel diameter 20 mm, and slope to drains 2%).

The above analysis shows that geotextile clogging is the main cause of the increased water level above the drainage system. Figure 8 shows the degree of saturation (i.e., ratio of moisture content to saturation moisture content) both in the waste layer and drainage layer. Since the hydraulic conductivity of geotextile decreases markedly, the geotextile layer acts as an aquiclude (about 10^−10^ m s^−1^), which enabled the accumulation of leachate above the geotextile layer and the fully saturated water level was more than 0.71 m. In addition, it should be noted that the porosity of the waste layer was set to a fixed value in this study. However, in real MSW landfills, the porosity and hydraulic conductivity of sub-layer waste decreases as a result of compaction [43], which would be likely to cause a further increase in the leachate head in the waste layer and more negative effects concomitantly.

Contrarily, when the drainage structure is composed of only a gravel drainage layer, the leachate in the waste layer might drain better. However, due to the increased hydraulic load of the leachate and mass loading of particulate matter near the outfall, the hydraulic conductivity in this area decreases and engenders a leachate mound at a maximum height of 21.2 cm. If taking the other two significant clogging mechanisms of microbe growth and Ca^2+^ precipitation into consideration as well, the LCS would fail in 5.5 years (to be discussed in next paper). At that time, the leachate head in it could be higher than 0.3 m, which would exceed the limit value of Chinese national standard GB 16889-2008 (Standard for Pollution Control on the Landfill Site of Municipal Solid Waste). Therefore, a dilemma appears that the quickly-clogged geotextile as a water-resistant layer impedes the downward flow of leachate and rising water level in the waste layer, but abandoning it would accelerate the gravel drainage layer clogging and increase leachate leakage.

**Figure 9:**
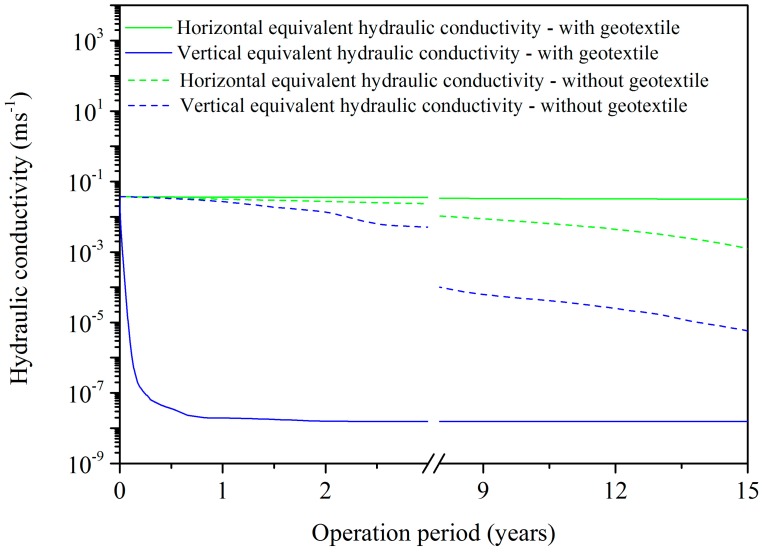
The changing of horizontal and vertical equivalent hydraulic conductivities of the LCS.

The horizontal and vertical equivalent hydraulic conductivities for the whole LCS govern the leachate movement in these two directions and further determine the leachate head above and in the LCS. To further analyze the clogging process, the geotextile and gravel layer were sliced into 18 and 47 layers in the vertical direction near the downstream end and the time-based changing of the horizontal and vertical equivalent hydraulic conductivities were calculated by the weighted and harmonic average of the hydraulic conductivity in each layer, respectively [44] (Figure 9).

The results showed that, within one year, the vertical hydraulic conductivity would drop to 10^−8^ m s^−1^ and the leachate drainage from the waste body could be prevented expeditiously if the geotextile was equipped as the filtering layer, while the horizontal hydraulic conductivity remained as high as the original value. Thus, the main problem of “water management” in a landfill is to drain more leachate and reduce the saturation in the landfill body rather than to lower the leachate head on the bottom liner. Without the geotextile, the horizontal and vertical equivalent hydraulic conductivities would decrease simultaneously, but the overall service life of the LCS could be prolonged.

By testing different geotextile thickness scenarios (Figure 5), a tendency could be seen for thicker geotextile to generate higher leachate head in the waste layer. Therefore, a feasible method applied to the dilemma is decreasing the geotextile thickness and making some particulate matter pass though the filter layer. In this way, the horizontal and vertical hydraulic conductivities might decline synchronously and a balance between the leachate mounding above and in the LCS could be achieved in a certain period. Periodically, a new LCS in the waste layer, such as a stratified drainage structure and vertical leachate wells, is required to be installed to replace the old clogged LCS.

## 5. Conclusions

In this study, the particulate matters in the raw leachate in China were characterized and the physical clogging development in LCSs and leachate accumulation in MSW landfills were analyzed. The results suggest that the MSW characteristics of high organic fraction and high moisture content result in the large quantity of leachate generation and the high concentration of particulate matter with larger size. These two features of leachate caused the rapid failure of LCSs, which is the main problem of “water management” in MSW landfills in China. Accordingly, MSW source separation, especially food waste diversion from landfill, could be necessary to delay the clogging development. Meanwhile, sewage sludge and demolition waste frequently ended up in landfills in China. As the fine particles accumulated, they might have been a notable source of TSS and accelerated the clogging process. Thus, disposal of sewage and demolition waste in MSW landfills should be banned.

The geotextile, as the upper layer of the LCS, effectively reduces the concentration of particulate matter entering the gravel layer and enables the gravel layer to maintain good permeability for a relatively long time. Therefore, to drain more leachate into the LCS and to lower the water mound in the landfill body is more important in landfills in China rather than to maintain the leachate head on the bottom liner within 30 cm. A feasible scheme is drilling some vertical drainage wells to conduct the accumulated leachate into the LCS. Because the geotextile above the LCS will be an impermeable layer for short periods, the role of geotextile should be balanced to optimize the LCS. The periodical installation of LCSs for every two or three lifts could be an option for leachate drainage in MSW landfills in China.

## Figures and Tables

**Table 1:** Primary properties of leachate from the Xiaowuji municipal solid waste (MSW) transfer station.

COD (mg L^−1^)	TSS (mg L^−1^)	VSS (mg L^−1^)	FSS (mg L^−1^)	Sediment Moisture Content (%)
28,105.7 ± 976.0	2262.2 ± 126.5	1960.0 ± 124.5	302.2 ± 46.3	84.8 ± 0.5

## Supplementary Materials

The following are available online at http://www.mdpi.com/1660-4601/15/2/318/s1, Figure S1: Hydraulic conductivity of geotextile under different filtration fluxes, Table S1: Particle diameter and concentration of each particulate matter size segment, Table S2: List of symbols.
