# Supplementary material for: The Physical Clogging of the Landfill Leachate Collection System in China: Based on Filtration Test and Numerical Modelling
Source: Int J Environ Res Public Health. 2018 Feb 12;15(2):318. doi: 10.3390/ijerph15020318 (PMC5858387; doi:10.3390/ijerph15020318)
Supplement: Supplementary file 1 [file ijerph-15-00318-s001.pdf]

Table S1. Particle diameter and concentration of each particulate matter size segment

Segment number	1	2	3	4	5	6
Diameter range (μm)	65–150	30–65	15–30	7.0–15.0	1.6–7.0	0.4–1.6
Representative particle matter diameter (μm)	107.5	47.5	22.5	11.0	4.3	1.0
TSS (mg/L)	63	242	364	433	768	399

Table S2. List of symbols

Variables and Coefficients	Illustration	Dimension	Value
A	van Genuchten parameter	L^{-1}	1 m^{-1} for waste and geotextile; 57.4 m^{-1} for gravel
a	geotextile thickness	L	2 mm
A_s	Happel correction factor	dimensionless	Calculated
C	particulate matter concentration	ML^{-3}	Calculated
$C_{(\theta)}$	water capacity	L^{-1}	Calculated
C_s	sedimentary particulate matter concentration	ML^{-3}	Calculated
D	hydrodynamic dispersion coefficient tensor	L^2T^{-1}	10^{-9} m^2 s^{-1}
dg	gravel diameter	L	Calculated
dg_0	initial gravel diameter	L	20 mm
h	pressure head	L	Calculated
H_p	posed in terms of pressure head	L	Calculated
I	particulate matter source or sink	$ML^{-3}T^{-1}$	1 m^3 m^{-2} $year^{-1}$
$K_{(h)}$	non-saturated hydraulic conductivity tensor	LT^{-1}	Calculated
K_0	initial hydraulic conductivity	LT^{-1}	10^{-4} m s^{-1} for waste; 10^{-3} m s^{-1} for waste; 0.053 m s^{-1} for gravel
K_a	coefficient for the hydraulic conductivity	LT^{-1}	9.8×10^{-6} m s^{-1} (n > 0.21); 2.4×10^{-8} m s^{-1} (n < 0.21)
K_b	coefficient for the hydraulic conductivity	dimensionless	22.9 (n > 0.21); 51.0 (n < 0.21)
K_s	saturated hydraulic conductivity	LT^{-1}	Calculated
K_t	real-time hydraulic conductivity	LT^{-1}	Calculated
M	van Genuchten parameter	dimensionless	0.5
N	van Genuchten parameter	dimensionless	2 for waste and geotextile and 2.44 for gravel
n	porosity	dimensionless	Calculated
n_0	initial porosity	dimensionless	0.375 for waste and gravel; 0.9 for geotextile
N_G	gravitational group number	dimensionless	Calculated
N_{Lo}	London parameter	dimensionless	Calculated
N_{Pe}	Peclet group number	dimensionless	Calculated
N_R	interception number	dimensionless	Calculated
S_e	effective saturation	dimensionless	Calculated
t	time	T	
v	pore-velocity	LT^{-1}	Calculated
W	water source	T^{-1}	Calculated
x	horizontal distance	L	
X_p	real density of particulate matter	ML^{-3}	1300 kg m^{-3}
z	vertical distance	L	

ε_p	inside porosity of packed particulate matter	dimensionless	0.85
η_f	Retained ratio of the particulate matter in the geotextile layer	dimensionless	Calculated
η_g	single-collector efficiency	dimensionless	Calculated
θ	pore-water content	dimensionless	Calculated
θ_r	residual water content	dimensionless	Calculated
θ_s	saturated volumetric water content	dimensionless	Calculated
λ	adsorption coefficient	L^{-1}	Calculated
λ_f	adsorption coefficient in geotextiles	L^{-1}	Calculated
λ_g	adsorption coefficient in gravel	L^{-1}	Calculated

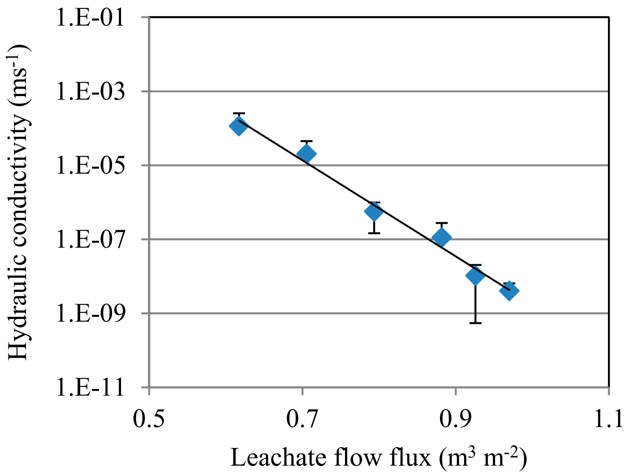

Figure S1. Hydraulic conductivity of geotextiles under different filtration fluxes
